# Understanding Metabolic Flux Behaviour in Whole-Cell Model Output

**DOI:** 10.3389/fmolb.2021.732079

**Published:** 2021-12-17

**Authors:** Sophie Landon, Oliver Chalkley, Gus Breese, Claire Grierson, Lucia Marucci

**Affiliations:** ^1^ BrisSynBio, University of Bristol, Bristol, United Kingdom; ^2^ Department of Engineering Mathematics, University of Bristol, Bristol, United Kingdom; ^3^ Bristol Centre for Complexity Science, Department of Engineering Mathematics, University of Bristol, Bristol, United Kingdom; ^4^ School of Biological Sciences, University of Bristol, Bristol, United Kingdom; ^5^ School of Cellular and Molecular Medicine, University of Bristol, Bristol, United Kingdom

**Keywords:** whole-cell modelling, machine learning, networks, snorkel, time series, weak learning

## Abstract

Whole-cell modelling is a newly expanding field that has many applications in lab experiment design and predictive drug testing. Although whole-cell model output contains a wealth of information, it is complex and high dimensional and thus hard to interpret. Here, we present an analysis pipeline that combines machine learning, dimensionality reduction, and network analysis to interpret and visualise metabolic reaction fluxes from a set of single gene knockouts simulated in the *Mycoplasma genitalium* whole-cell model. We found that the reaction behaviours show trends that correlate with phenotypic classes of the simulation output, highlighting particular cellular subsystems that malfunction after gene knockouts. From a graphical representation of the metabolic network, we saw that there is a set of reactions that can be used as markers of a phenotypic class, showing their importance within the network. Our analysis pipeline can support the understanding of the complexity of *in silico* cells without detailed knowledge of the constituent parts, which can help to understand the effects of gene knockouts and, as whole-cell models become more widely built and used, aid genome design.

## Introduction

Recent years have seen a significant increase in the availability of high-throughput biological data (Gomez-Cabrero et al., 2014). The integration of data from methods that are becoming cheaper and more accessible (Wetterstrand, 2010) reveals interactions between cellular processes (Manzoni et al., 2018), aiding analysis (Zampieri et al., 2019). Leaps in the scale and capabilities of biological modelling give great scope for *in silico* data generation, and though mathematical models cannot fully replicate living cells, their output can help to understand biological mechanisms and inform experimental design to improve *in vivo* data collection. These models can formalise processes at a specific level (e.g., translation) or construct a trans-omic network of the relationship between different cellular processes (Yugi et al., 2019) and couple metabolism with gene expression (O’brien et al., 2013). Whole-cell models simulate every cellular process throughout the life cycle of a cell—only two are published, which model the life cycle of *Mycoplasma genitalium* (Karr et al., 2012) and *Escherichia coli* (Macklin et al., 2020). We focus on the *M. genitalium* model. This consists of 28 submodels that use multiple mathematical methods (linear programming and differential equations) to represent processes such as metabolism and cytokinesis, which integrate together at every time step.

The model is highly complex, is computationally expensive, and generates huge amounts of time series data relating to thousands of variables. Interpreting whole-cell model *in silico* data can be difficult, but large-scale analysis is possible. Tools are required to automatically process and consolidate the output so they can be viewed and clarified, even by those with little computational expertise. Existing software tools that visualise whole-cell model output (Lee et al., 2013; Karr and Pochiraju, 2018) have limited capacity for processing large and varied datasets—they focus on visualisation of different output streams, so all analyses are done by eye, and there is no dimensionality reduction or statistical methodology.

A whole-cell model, with appropriate analysis software to process its output, could be a powerful predictive tool for gene editing. Genetic modifications can be trialled in a model before being physically made to save time and resources, and whole-cell models can be coupled with algorithms to predict genetic modifications intended to produce a chosen phenotype (Haimovich et al., 2015). Machine learning methods are suitable for whole-cell model analysis as they are data-driven, so they can identify correlations and classify data with few assumptions and little biological knowledge. Metabolism is one of the most widely modelled cellular subsystems; a stoichiometric matrix is used to create a constraint-based metabolic model (CBM), which can be used to predict steady-state fluxes (Bordbar et al., 2014). There have been applications of machine learning to CBMs, consolidated by Zampieri (Zampieri et al., 2019). Many have coupled CBMs with discriminative classifiers (Ho, 1995; Noble, 2006; Yegnanarayana, 2009), to predict or classify gene essentiality, drug side effects, and protein functions. Others have used unsupervised learning to explore patterns and pathways in metabolic systems (You et al., 2006). These methods of prediction and analysis can be scaled to whole-cell models. However, whole-cell model output is composed of time series—contrary to CBM output, which is steady-state rates—and the labelling of these types of data is becoming a barrier to large-scale machine learning. As computational power increases and new data analysis algorithms are developed, the availability of fully labelled datasets to train and validate models is a limiting factor, and so new methods are being formed to automatically label data.

Time series data come from all physical systems. Difficulty in interpreting it arises from the importance of ordering of different events, meaning that attributes of the data are dependent on each other in complex ways (Hannan, 2009). Of the various machine learning methods for time series classification, deep learning has emerged as the most reliable (Wang et al., 2017; Fawaz et al., 2019), although accuracies of each method vary with different datasets. There are also other factors that affect the performance of an algorithm, such as feature selection, feature engineering, and data pre-processing.

Many of these methods are supervised, meaning that they require labelled data in order to train a model. Historically, these labels would be manually generated by an expert to capture the ground truth of the problem, but labelling data manually is time-consuming and unfeasible for huge datasets. A solution to this problem is weak supervision, which uses weak labels (that do not express the ground truth) created from a model designed to map labels onto instances of the data (Zhou, 2018). Snorkel is a methodology that creates a generative model (a statistical model of the joint probability of a variable and target label) to automatically produce weak labels, after collating metrics from multiple manually defined labelling functions using features from the data (Ratner et al., 2019; Ratner et al., 2017).

Feature extraction is one of the most important aspects of building a machine learning model and can be the difference between failure and success (Domingos, 2012). It is also generally based on expert knowledge about the physical system (Barandas et al., 2020), as the most relevant features for analysis will vary depending on the objective of the machine learning model and the behaviour of the time series. The issue of time series analysis of whole-cell model generated metabolic flux is that there is very little experimental data for dynamic flux in bacterial cells, so the features that best define the flux behaviour are not intuitive. There has been previous work on dynamic metabolic fluxes, where reactions rates were calculated from derivatives of measured external metabolite concentration, or using dynamic metabolic flux analysis (DMFA) (Kuriya and Araki, 2020). For DMFA, a metabolic flux analysis process was used to minimise the sum of squared residuals between the actual and predicted flux rates. Then, the DMFA process was used to fit linear functions between consecutive time points. The methods were computationally inexpensive, due to the linear fit, and it was found that a lower number of time points produced a fit with smaller confidence intervals, suggesting that linear fits are suitable for approximating metabolic fluxes. Another method used dynamic flux balance analysis (dFBA) and polynomial fitting to find functions for reaction rates (Leighty and Antoniewicz, 2011). Polynomial functions were fitted to experimental data from metabolite concentrations, which were then differentiated to find functions for growth rate. These were used as boundaries for dFBA, enabling accurate simulations of reaction behaviour in time. Both of these methods deal with relatively smooth data, and estimation of fluxes from concentration derivatives also involves a smoothing process, which results in loss of information (Lequeux et al., 2010). As some of the flux behaviour we see from the *M. genitalium* model oscillates significantly in time (as in Supplementary Figure S1), to analyse this, we must extract features that can capture some of the variation. Analysis of oscillatory time series is relatively common, but this is usually within the context of understanding the physical system—for example, oscillatory time series decomposition has been carried on the phase dynamics of well-understood systems (Matsuda and Komaki, 2017).

It is important to consider that most machine learning algorithms are treated as black boxes, so results are created without context. For explanations of the functions of underlying structure in complex systems, network science can be used (Gosak et al., 2018). Network science is an area that has long been applied to the analysis of biological systems: protein interactions, metabolic reactions, and transcription regulation can be formalised as networks, leading to discoveries regarding properties of their interactions (Barabasi and Oltvai, 2004). Network structure has been used to predict metabolic functions and find pathways for metabolite flow (Stelling et al., 2002) and to find control loops within gene networks (Wong et al., 2012).

The complexity of genomic interactions, even in cells as small as *M. genitalium*, is such that there is not a clear path from the genome after knockouts to the end phenotype. Even with functional annotations, the genomic context of the genome (which will be several hundred genes after a single gene knockout) cannot be disregarded, as there may be redundancy in the genome, or unprecedented gene product interactions. The removed gene/s will not tell the full story, but zooming out to examine a large set of different genotypes through their metabolic fluxes can show us the trends across the full set of knockouts, providing a different angle than that of focusing on a single gene.

Here, we present a novel analysis pipeline that combines whole-cell model simulations of wild-type and gene knockout cells with time series classification and network analysis. The main steps include automatic labelling of metabolic fluxes as normal or abnormal (where normality refers to the behaviour of a reaction flux from a knockout simulation with respect to the behaviour of that reaction in a wild-type simulation), dimensionality reduction of the reactions for visualisation, and network analysis of the reactions. This analysis—looking at intermediate steps that connect genotype to phenotype—aims to increase our understanding of cellular processes and provides foundations for *in silico* genome design.

## Materials and Methods

### Description of the Data

We began with two sets of data—one to train the machine learning models and one to apply them and analyse the output. The simulations were generated from running the *M. genitalium* whole-cell model on a supercomputer cluster, with each gene singly knocked out. The model requires 8 GB of RAM for each simulation and was run on BlueGem, a 900-core supercomputer at the University of Bristol, using MATLAB R2013b. It is available at https://github.com/CovertLab/WholeCell. The raw metabolic flux time series was then converted to Pandas DataFrames and stored in a pickle format to save space. The training set consisted of time series of reaction fluxes for three repetitions of every possible single knockout from the *M. genitalium* model, of which there are 359, plus 200 wild-type simulations. Each time series is 50,000 s in total, and we used the time series of 279 reactions from each simulation. There was 1,270 simulations in total. The dataset that we applied to the analysis consisted of 10 repetitions of all of the single gene knockouts, with the same reaction time series, and so this dataset has 3,411 simulations in total. One knockout, MG_469, consistently caused the model to crash and the simulations to terminate, and a few simulations did not complete due to errors on the supercomputer cluster. The metabolic flux data are about 200 Mb per simulation after processing, so the training dataset (three repetitions of each single knockout) is ≈200 Gb, and the analysis dataset ≈700 Gb. More repetitions of each knockout would make for a more accurate dataset, but due to the size of the data, we were limited by storage space.

### Labelling

Snorkel is a system that takes input data points and manually defined labelling functions and collates these into a generative model that outputs probabilistic labels for the data. The labelling functions will produce noisy labels, which are then used as weak supervision for a stronger predictive function by combining three measures—the labelling propensity (whether the data point has been assigned a label or not), the accuracy of each label, and the correlations of the multiple labelling functions. The label matrix generated from these measures is then used to define an exponential distribution that can predict probabilistic training labels. The normality of 10 reactions was manually labelled by visual inspection of the time series, comparing features of the plots such as smoothness and linearity with wild-type time series from the same reactions (Correll et al., 2012; Correll and Heer, 2017), and used to validate Snorkel’s weak labels, the accuracies of which are shown in Table 1. The algorithm was implemented using the Snorkel library in Python.

**TABLE 1:** Accuracies of Snorkel’s weak labels for 10 manually labelled reactions.

Accuracy	
Aas4	99.6%
AceE	99.1%
Adk3	90.2%
Apts_Asp	95.8%
Apts_Trp	83.1%
ArcC	77.8%
DcdK	97.0%
Pyk_DADP	85.0%
Pyl_GDP	69.2%
TX_AROP22	94.3%

The manual labelling was done based on the phenotypic classes defined by the original publication of the *M. genitalium* model, which used the production capacity of various features from the model output to classify a simulation (Karr et al., 2012). The combinations of these features that contribute to a particular class are detailed in Table 2, and the simulations used in the analysis dataset were all labelled by manual inspection of the model output.

**TABLE 2:** Manual labels of phenotypic classes (shown on the left-hand column) and their corresponding combinations of substance production (the column headings).

	DNA	RNA	Protein	Growth	Division
Metabolic	×	×	×	×	×
RNA	*✓*	×	×	×	×
Protein	*✓*	*✓*	×	×	×
Slow growing	*✓*	*✓*	*✓*	*✓*	×
DNA	×	*✓*	*✓*	*✓*	×
Septum	*✓*	*✓*	*✓*	*✓*	×
Non-essential	*✓*	*✓*	*✓*	*✓*	*✓*

Note. A cross means that there is no active production of that substance in the case of DNA, RNA, and protein; and in the case of growth and division, these things do not occur. A tick means that opposite—so, for example, in a simulation classified as “non-essential,” we see production of DNA, RNA, and protein, as well as both growth and division; and in a simulation classified as “metabolic,” we see none of these things. In the case of the “slow growing” phenotype, division begins at the end of the simulation but does not complete.

### Training and Tuning the Neural Networks

Once the data are fully labelled, a standard discriminative model can be trained for classification. In this case, we chose to use a neural network, implemented with the Python library tensorflow (version 2.0.0-rc0). With the use of the data labelled by the generative model, a neural network was trained for each reaction. Each neural network had four hidden layers and used a softmax activator function and Adam optimiser. Different combinations of hyperparameters (epoch size, batch size, and number of nodes in a layer) were tested, so that an optimal combination could be used for each network to find the highest accuracy. Generally, the combination of hyperparameters can have a significant effect on the neural network output, so these factors are important. Epoch size refers to the number of rounds of back-propagation performed by the network, batch size means the number of training data samples input before the model updates, and number of nodes refers to number of nodes of the network in each hidden layer. Epoch size will leave the data underfitted if too small and overfitted if too large; batch size is generally optimised for processing time (in that larger batch sizes will train the network faster, whereas a smaller batch size may help the weights converge faster); and the number of nodes is usually taken to be some number between the amount of input nodes and the amount of output nodes. There is no set method for selecting hyperparameters for neural nets, and it is frequently taken to be a trial-and-error process (Sarle, 1994). We tuned our neural networks *via* a brute-force approach, where different parameters within a set range were trialled to increase the accuracy of the network. Epoch size was kept relatively low; as after some testing, many of the neural networks converged to accuracies > 95% after only five epochs, and so we tested epoch values of 5, 10, and 15. Batch sizes of 50, 100, and 150 used, and node numbers of 750, 1,500, and 2,250 were tried, where we selected the network with hyperparameters that gave the highest accuracy. The reactions from neural networks that gave accuracy of less than 70% were removed, leaving 267 reactions and neural networks with a mean accuracy of 93.6%. K-fold cross-validation was performed to check if overfitting was an issue, using the sklearn Python library (version 0.21.3), with 10-fold. The accuracies across the folds are shown in Supplementary Figure S2 and averaged across the folds for each reaction. As the averaged accuracies across the folds do not differ significantly from accuracies recorded, we conclude that the data have not been overfitted.

### Network Formation and Features

After the neural networks were trained and fluxes classified across the dataset, we turned to network analysis. With the stoichiometric matrix for the metabolism, **S**, taken from the *M. genitalium* model knowledge base, we reduced it to its binary format (as we were focusing on the topology of the metabolic network rather than the exact stoichiometry) to form a metabolic adjacency matrix **A** from the relationship

$$A=S^{t}S,$$ (1)

which can create a widely used graphical representation of a metabolic network, where the reactions form nodes of the graph, and the substrates form edges that connect them (Palsson, 2006). We were able to find a set of driver nodes (the set of nodes that must be controlled in order to fully control the network) using the maximal_matching function in Python’s NetworkX library (version 2.4). This function takes an undirected graph and greedily finds a matching by iterating over pairs of edges in the graph to see whether the node that connects them is in the matching. The pathways associated with the driver nodes were found *via* the Enzyme Commission numbers from the supplementary material of the *M. genitalium* model (Karr et al., 2012), where the Python library bioservices was used to look up the pathways for each EC number from Kyoto Encyclopedia of Genes and Genomes (KEGG).

The metabolic sub-networks were plotted in python-igraph where, for each class across the dataset, the affected reactions are shown as a sub-network with a colour gradient corresponding to how frequently that reaction behaves abnormally. A threshold for “noisy” reactions was found from wild-type simulations, where an exponential distribution was fitted to the frequencies of reactions classified as behaving abnormally by the neural networks. For a wild-type simulation, in theory, all reactions should be classified as normal, but as the *M. genitalium* model is stochastic, there can be a range of different behaviours, depending on the initial conditions of the simulation and other random processes (e.g., radiation and DNA damage). The interval under which 95% of the data were contained was found, and this value was selected as a rate parameter, which was used as the threshold of significance for whether a reaction was considered to be behaving abnormally consistently.

We then performed principal component analysis (PCA) using the SciPy library (version 1.3.1) to reduce the data to two dimensions and plot the data on a scatter plot using Seaborn (version 0.9.0). After the reduction, 84% of the variance in the full dimensions of the data was conserved, so there was no significant information loss after this operation. After having found the driver nodes, we trained a linear support vector machine (SVM) for the normality of each one to separate the data points on the PCA plot, selecting those that could divide the data with > 95% accuracy. For the SVM, we used the sklearn Python library (version 0.21.3).

## Results

**FIGURE 1:**
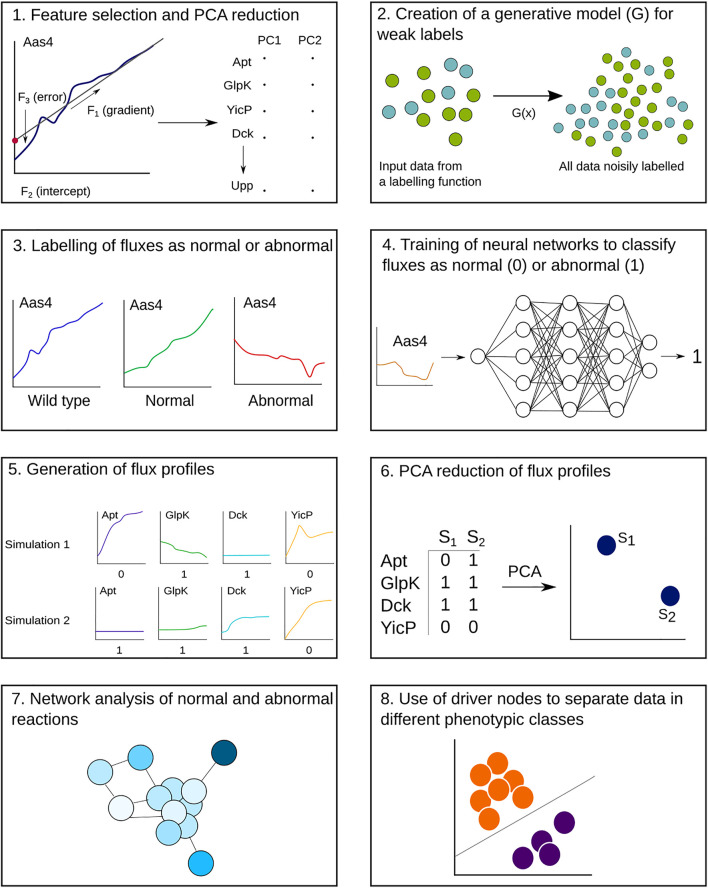
Step-by-step workflow of the analysis pipeline, beginning with the metabolic fluxes from the whole-cell model output. Steps 1–4 are applied to a training dataset of gene knockout simulations, where the end result is a trained neural network for each reaction. (1) For each reaction flux time series, four features are extracted and reduced to two dimensions through principal component analysis (PCA). (2, 3) The extrema of these data are used to define boundaries for normal and abnormal behaviours, which are then used to create a generative function to map labels onto the reactions. (4) Neural networks are trained using these labelled data to classify reactions as normal or abnormal. Steps 5–8 are applied to a separate analysis dataset of gene knockout simulations. (5) The neural networks are used to classify the analysis dataset and create a flux profile for each simulation. (6) The flux profiles are reduced to two dimensions and plotted. (7, 8) Network analysis of the reactions reveals nodes that control the metabolic network and correlate with different phenotypes after gene knockouts.

A schematic of our pipeline is shown in Figure 1, with the main steps of weak labelling, neural network classification, and network analysis shown. We began with two datasets: one for training and testing the neural network and one for analysis of single knockouts. The training dataset contained three repetitions of all 359 possible single gene knockouts, plus 200 wild-type simulations, giving 1,270 simulations in total. Each simulation had 279 dynamic reactions out of the total 645 (over half of the reactions were consistently at steady state throughout the cell life cycle, which does not require a complex classifier to identify), with up to 50,000 timesteps. Although the exact steady-state values may vary across simulations, we focused specifically on the reactions that have behaviours that change in time, assuming that they are more likely to show the most sensitive components of the metabolism. Given that metabolic networks are formulated with steady-state behaviours in mind, reactions that deviate from this seemed to be the most interesting to analyse, with regard to understanding the cell phenotype. The analysis dataset consisted of 10 repetitions of the 359 gene knockouts, with the same number of reactions and timesteps, totalling 3,411 simulations. There are some gaps in the dataset, as some files were corrupted, and one knockout consistently caused the model to crash.

The *M. genitalium* whole-cell model has drastically varying fluxes through different reactions (see Supplementary Figure S1). Furthermore, it is not always clear how the removal of a particular gene will affect cellular processes or cell viability. For each reaction, we presume there is a range of normal behaviours over which the cell can produce all necessary compounds for division, and dynamics outside of that range result in negative effects (e.g., build-up or depletion of certain metabolites) that affect the rest of metabolism and disrupt other processes, potentially causing cell death. The normality of reaction fluxes in a simulation can be used to understand the effects of gene knockouts through the cell cycle, and how metabolism is affected. This can help with predicting and explaining the effects of gene knockouts and looking at patterns across different simulations. We visualised the reaction flux behaviour across our entire dataset, and we looked at the topology of the metabolic network (in particular, how the network can be controlled by input nodes) to help explain the role of different reactions.

### Implementation of Snorkel for Weak Labelling

Manual labelling was impractical with such a large dataset, so we implemented Snorkel, which has previously been shown to perform as accurately as hand labelling (Ratner et al., 2019). There are other methods of weak supervision available, but they use either inaccurate labels (which still require a manually labelled dataset) or locate incorrect labels within a previously labelled dataset Northcutt et al. (2019). Inaccurate labels are those that are known to be incorrect, and imprecise labels are those that contain some high level information about the data, but do not show the ground truth. Snorkel is the main approach that uses imprecise labels for time series (Robinson et al., 2020), as other approaches have used imprecise labels for semantic similarity in words, which is not applicable to time series (Saunshi et al., 2019).

Snorkel requires manually defined labelling functions, which are an important heuristic for the basis of the methodology. The underlying patterns are used to form probabilistic labels, so together they need to capture some approximation of ground truth. In this case, we created labelling functions by amalgamating four key features extracted from each reaction flux time series. There is very little information in the literature about what normal behaviours for metabolic fluxes should look like, so we must make an assessment of the most important features from time series inspection.

**FIGURE 2:**
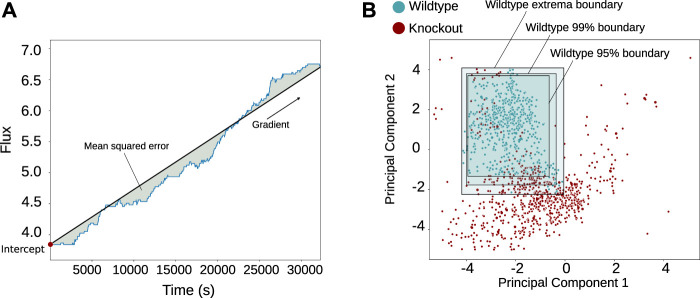
Method of feature extraction and normality classification shown graphically. The features shown in plot **(A)** were taken, and principal component analysis (PCA) was applied for each reaction flux in each simulation across the dataset to create plot **(B)**, retaining 82.9% of the variance. The wild-type simulations are shown in blue, and then three boundaries are shown (extrema, 99% confidence intervals, and 95% confidence intervals), which are used to form three labelling schemes.

As Snorkel is designed to work with noisy and sometimes conflicting labels, we used a simple method to define the labelling functions. A linear regression function was fitted to each time series; and the intercept, gradient, coefficient of determination (*R* ^2^), and mean squared error were found (Figure 2). These captured the variation observed and shown in Supplementary Figure S1: smoothness/oscillation in the mean squared error, the increasing or decreasing nature in the gradient, and the linearity in the coefficient of determination. These were features that we chose based on manual inspection of the reaction behaviour, with the intent of describing the important aspects of the time series, so in choosing them we aimed to capture the most relevant information. Fitting non-linear functions to the data may have provided more accurate labelling functions, but due to the complexity and variety of the time series, this would have required a many visual analyses and likely a broad set of different non-linear functions.

The results were reduced through PCA (where 82.9% of the variance was conserved across the reactions), leaving a two-dimensional space over which boundaries of different thresholds could be drawn, which was much simpler and faster to visualise and compute in two dimensions than it would have been before the dimensionality reduction (Figure 2). Using two dimensions allowed us to easily verify visually the efficacy of this labelling method while approximately dividing the data for the weak labelling. Loosely, the boundaries were defined by the extrema of the wild-type simulations, which were taken to be the edges of normal behaviours for each reaction (Figure 2). Any simulations outside these boundaries were classified as abnormal. Other shapes could also be used at this stage.

Three different boundaries were defined for different labelling schemes, as different confidence thresholds performed better or worse depending on the reaction. Boundaries at the extrema and then at 99% and at 95% were selected as the three labelling functions after comparison of their performance and then combined to form the generative model. We then implemented Snorkel, leaving us with 1,270 weakly labelled time series for each reaction. Ten reactions were manually labelled as normal or abnormal to test the accuracy of Snorkel’s labels, where characteristics like smoothness or the increasing or decreasing nature of the time series were used as comparison features to decide whether the behaviour of a reaction was normal or abnormal. The majority of the Snorkel labels gave over 90% accuracy, with the lowest at 69.2% (see the *Materials and Methods* and Table 1).

### Training of Neural Networks and Flux Profiling

The Snorkel results were used to train a neural network for each reaction, as artificial neural networks are some of the most effective classification algorithms (Caruana and Niculescu-Mizil, 2006; Raczko and Zagajewski, 2017). Neural networks consist of layers of nodes, representing artificial neurons with assigned weighted connections. The weights are adjusted through rounds of backpropagation or epochs until they predict correct classes for different types of input (Kröse et al., 1993).

**FIGURE 3:**
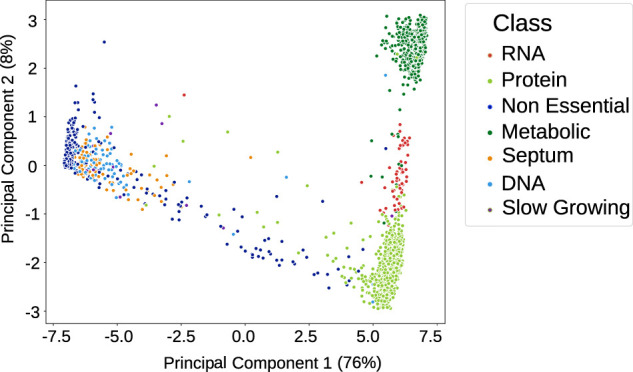
Principal component analysis (PCA) plot of the flux profiles (binary strings of normal vs. abnormal classifications for each flux in a simulation) from 3,411 gene knockout simulations, reduced to two dimensions (while retaining 84.5% of the variance) and then shown in different colours that correspond to manual labels. The classes are defined by the presumed root cause of lack of cell division; or if the cell divides, the simulation is classified as non-essential.

Once trained and assessed for accuracy using k-fold cross-validation to verify that they had not been overfitted (see the *Materials and Methods* section and Supplementary Figure S2), the neural networks were used to classify the normality of reactions for the analysis dataset. From this, we generated a “flux profile” for each simulation: a binary string for each reaction within that simulation, where 0 means normal behaviours and 1 means abnormal. Reactions for neural networks with less than 70% accuracy were removed (of which there were 12 in total), leaving 267 reactions with a mean accuracy of 93.6%. We applied PCA to reduce the flux profiles to two dimensions while retaining most of the variance and visualised, as shown in Figure 3. Each point is the flux profile of a simulation, and the principal components correspond to the reduced dimensions of the reaction flux profiles. As PCA preserves global and pairwise distances between all data points, unlike other dimensionality reduction processes that focus on local distance (such as t-Distributed Stochastic Neighbor Embedding (Van der Maaten and Hinton, 2008) and Uniform Manifold Approximation and Projection (McInnes et al., 2018)), this enables us to see not only the relationship between data points but the relationship between the different clusters, leading to clearer interpretability.

The analysis dataset simulations were previously hand-labelled by phenotype according to differences in cell behaviours of the simulation output. The labelling classes were non-essential, DNA disruption, RNA disruption, metabolic disruption, protein disruption, or septum disruption (Rees-Garbutt et al., 2020)—see the *Materials and Methods* for details. The non-essential class is defined by whether the cell divides or not, in keeping with current definitions of gene essentiality (Zhang and Zhang, 2008), and the other classes are defined by what is indicated by the output data to be the root cause of cell death.

In Figure 3, several clusters of flux profiles are visible. To validate their significance, we coloured the flux profile points according to manual labels of the phenotype that has occurred after the knockout; it can be seen (Figure 3) that these clusters correspond to the manually defined phenotypic classes. This suggests (intuitively) that different sets of reactions behave abnormally for each different class of phenotype, with different scales in the proportion of reactions affected, which will separate the different classes in the PCA space. We expect the majority of reactions in a simulation labelled as non-essential to be classified as normal and the non-essential simulations to be clustered together in the PCA space, as their flux profiles will be similar. Then, for simulations with greater disruption (e.g., the metabolic phenotypic class, where there is no growth and no DNA, RNA, or protein is created (see the *Materials and Methods* section), where many reactions are behaving abnormally), these will be placed much further away from the non-essential cluster.

### Analysis and Biological Context Within the Metabolic Network

The clustering analysis is useful to show the big picture across the entire dataset but does not suggest much biological insight that could be applied to lab experiments. In order to make sense of the data in a way that can be used in an experiment, we need to understand these results at the scale of groups of genes or reactions. To ascribe biological meaning to trends seen across the dataset, we analysed the topology of the metabolic network, as this is a representation of the relationships between different reactions, and so we can see how it is affected by reactions behaving abnormally after knockouts. It has been shown that the modularity of the *E. coli* metabolic network corresponds to metabolic functions (Ravasz et al., 2002), and so, from a graphical perspective, we aimed to explain some of the biology behind the phenotypic classes and the flux profiles. The *M. genitalium* metabolic network is significantly smaller than many bacterial metabolisms (645 reactions vs., e.g., 2,382 in *E. coli* (Feist et al., 2007)), due to its genome size—however, analysis is not trivial. We used a graphical representation of the network, where each reaction is a node, and substrates that connect reactions are edges, as in the stoichiometric matrix of the metabolism in the knowledge base of the *M. genitalium* model. We visualised the reactions affected across each class in individual graphs, shown in Supplementary Figure S3.

There are multiple ways to gauge the importance of a node within a network. Most commonly used are centrality measures (Freeman, 1977), but for dynamic networks, we can focus on the control of the network *via* the nodes. From the graphical representation, we used a maximal matching algorithm to find driver nodes. Driver nodes are the set of nodes in the network that need to be managed in order to have full control over the system, which can be found for both directed and undirected networks (Liu et al., 2011; Nacher et al., 2019)—therefore, in terms of input into the metabolism and flow through the metabolic pathways, their behaviours affect other reactions downstream, and they could be indicators of phenotypes after gene knockouts. The driver nodes of the network are shown and named in Supplementary Figure S4. For each driver node, the pathways associated with that reaction were found from KEGG (Kanehisa and Goto, 2002) or (if there was no annotation for that reaction) the pathways associated with reactions that were one degree away from the driver, as shown in Table 3.

**TABLE 3:** List of all of the driver nodes, whether they can linearly separate different phenotypic classes in the PCA space, and their associated pathways (if available).

Driver	Pathways	Linearly separable
TX_CO2	Glycolysis, TCA cycle, pyruvate metabolism, carbon metabolism	Yes
TX_COA	Glycolysis, TCA cycle, pyruvate metabolism, carbon metabolism, pantothenate and CoA biosynthesis, methane metabolism	Yes
TXPYDX	Vitamin B6 pathway	Yes
TX_ACAL	Pentose phosphate pathway	No
TX_CAP	Purine metabolism, carbon metabolism	No
TX_DDCA	Glycerolipid metabolism	n/a
TX_FOR	One carbon pool by folate, carbon metabolism	n/a
TX_H2O2	n/a	Yes
TX_HDCA	Glycerolipid metabolism	Yes
TX_HDCEA	Glycerolipid metabolism	Yes
TX_LIPOATE	n/a	Yes
TX_NAC	Nicotinate and nicotinamide metabolism	Yes
TX_O2	Purine metabolism, pyrimidine metabolism	Yes
TX_OA	Pyruvate metabolism, carbon metabolism, methane metabolism	No
TX_OCDCA	Glycerolipid metabolism	Yes
TX_OCDCEA	Glycerolipid metabolism	Yes
TX_RIBFLV	Riboflavin metabolism, biosynthesis of secondary metabolites	Yes
TX_THF	One carbon pool by folate, folate biosynthesis	Yes
TX_TTDCA	Glycerolipid metabolism	n/a
TX_TTDCEA	Glycerolipid metabolism	n/a
Upp	Pyrimidine metabolism	Yes

Note. PCA, principal component analysis; TCA, tricarboxylic acid.

Metabolic networks are known to be robust (Smart et al., 2008; Holme, 2011), so many reactions can be individually removed without causing adverse effects. However, within *M. genitalium* metabolism, very few metabolites are organically synthesised (Dybvig and Voelker, 1996). Transport reactions for essential substrates such as amino acids are far more important than they might be in a larger cell that has the capabilities to synthesise these things itself. Within the metabolic network for the most widely used constraint-based *E. coli* model [iAF1260 (Feist et al., 2007)], 75% of the driver nodes are transport reactions, compared with 95% in the *M. genitalium* metabolic network.

**FIGURE 4:**
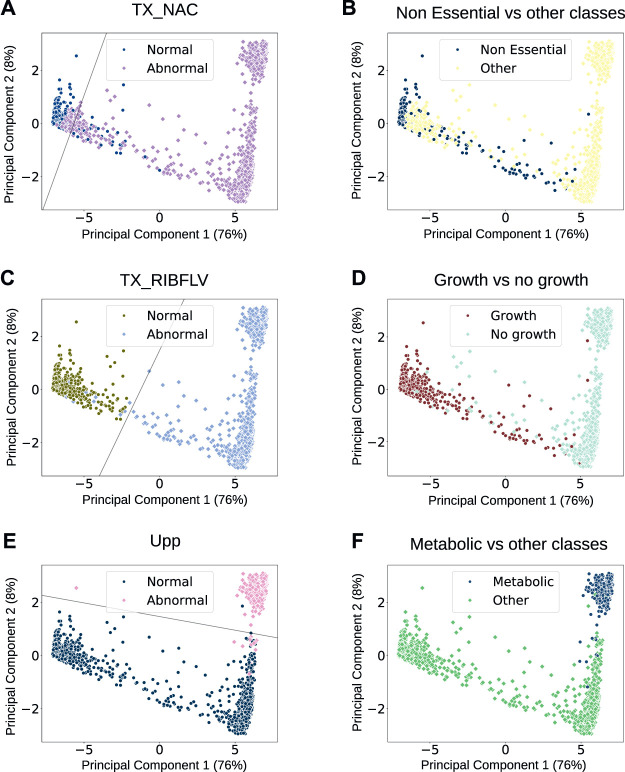
Scatter plots of all flux profiles reduced to a 2D feature space. On the left [plots **(A,C, E)**], each point is labelled with the behaviour (normal or abnormal) of a single reaction that is a driver node. The lines shown decision boundaries for support vector machines (SVMs); models that form a hyperplane to linearly separate different classes of data, where in this case the classes will be flux profiles where the specified reaction behaves normally, and flux profiles where the specified reaction behaves abnormally. The reactions are referred to using their identifiers that are used in the model. On the right [plots **(B, D, F)**], each point shows the manual label of the phenotypic classes that correlate with the behaviour of the reactions on the left—the simulations that show normal behaviours of TX_NAC have a 92% correlation with those that are manually classified as non-essential; those that show normal behaviours of TX_RIBFLV show a 95% correlation with those that are manually classified as non-essential, DNA, or septum phenotypes (which all show cell growth), and those that show abnormal behaviours for Upp show a 97% correlation with those that are manually classified as metabolic.

We found several driver nodes that can be individually used as features to divide the data into separate classes (referred to in the text using their reaction identifiers from the model). For all driver nodes, we modelled a linear SVM across the 2D data of the analysis dataset. We then selected those that could separate the data into normal vs. abnormal behaviours with over 95% accuracy as good and simple indicators of metabolic behaviours, shown in Figure 4. Of the driver nodes, 83% could linearly separate the data with greater than 95% accuracy (listed in Supplementary Table S4), compared with only 60% of the non-driver nodes, demonstrating their significance. Additionally, we can use individual driver nodes to mark phenotypic classes—normal behaviours for TX_NAC, the reaction that transports nicotinamide into the cell, correlate strongly with the simulations classified as non-essential, with a phi coefficient [a measure of correlation between binary variables (Ekström, 2011)] of 92%. Behaviours of TX_RIBFLV can split the dataset into the classes where we see growth (non-essential, septum, and DNA phenotypes) and the classes where there is no growth (metabolic, RNA, and protein phenotypes) with a phi coefficient of 95%. Equally, we can see that abnormal behaviours for Upp (dephosphorylation of uracil) are strongly indicative of a metabolic phenotype and can be used as a feature to separate metabolic disruption phenotypes from other types of phenotype, with a phi coefficient of 97%. Overall, the driver node analysis showed that it is possible to identify important reactions within the network that correlate with certain cell behaviours, meaning that we can focus on these to understand the end phenotype rather than the entire set of reactions.

## Discussion

We have shown multiple analysis methods that take a large high-dimensional dataset and distill it into visualisations that are easy to interpret. The pipeline of weak labelling followed by neural network classification is applicable to any system that outputs time series, although the features used for the initial labelled schema may have to be changed, according to what the researcher intends to look for, and the type of time series that is being analysed. As discussed previously, it is particularly useful for where “normal” behaviours for a system is not well defined, and the mechanisms that underlie the system cannot be distilled into a form that is understood. We have shown that it is applicable for black-box models, but it could also be used for data from complex physical systems where we do not understand the fundamental structure, such as meteorological phenomena. Additionally, the driver node analysis is applicable to any system where there is input, output, and internal structure, as it can highlight the most important parts of a high-dimensional system.

The processing of complex data is imperative to understand whole-cell model output, and this method demonstrates how the behaviours of specific reactions can be used as a marker of a particular phenotypic class and their importance to the corresponding cellular process.

Understanding the effects of single gene knockouts is a deceptively difficult task, as the domino effect of gene removal can cause large changes in the behaviour of a cell through its life cycle. Visualising and analysing thousands of time series is a challenge faced by many branches of research. These two problems come together in the context of whole-cell models. Using Snorkel and neural networks, we have been able to classify metabolic fluxes as normal or abnormal and visualise them in two dimensions, meaning that the dataset separates into groups that can be interpreted. Whole-cell model data must be understood in the context of controllable biological mechanisms to be relevant to genome design: in order to use knowledge gained from modelling in real cells, we must understand the internal operations as well as the output. The flux behaviour across different gene knockouts, and in particular the driver nodes, can show the links between genotype and phenotype, plus unprecedented effects that a gene may have on reactions seemingly unrelated to its functional annotation, on a scale that is only possible in a whole-cell model. As this analysis gives an overview of the entire metabolism, we can approach the problem of understanding gene knockouts in a way that includes the genomic context of the remaining genes and the behaviour of their associated reactions, rather than examining the phenotype with regard to the single gene that has been removed.

The driver nodes can also give insight into the essentiality of *Mycoplasma* functions. Most of the driver reactions are not associated with annotated genes, as many transporter proteins are putative—however, given that *M. genitalium* synthesises very few compounds and gains most from its surrounding media, this is an important knowledge gap. The external media for *Mycoplasma* culture is generally undefined rich media, so knowledge of exactly which of the media components are essential for growth would be valuable for lab use and simplify *Mycoplasma* production (Gaspari et al., 2020). This may also help with linking un-annotated genes with modelled functions, leading to better understanding of the *M. genitalium* genome. For example, an essential protein in JCVI-syn3A [one of the first synthetic organisms; designed to function as a minimal cell (Breuer et al., 2019)] has recently been classified as a riboflavin transporter protein, showing that vitamin transport is an essential function for a minimal organism (Zhang et al., 2021). As *M. genitalium* does not synthesise riboflavin, this suggests that one of its un-annotated genes must be a riboflavin transporter. As more wet lab work is done with *M. genitalium*, it will be interesting to compare it to the model results and the importance of different driver nodes. The essentiality of similar transport reactions could also be looked at in other organisms, as these results may be applicable to other *Mycoplasma*s.

For genome design, there has long been an idea of “modularity” in cells, at different scales and abstractions (Papin et al., 2004). Cellular subsystems that use a unique set of molecules and rules to perform a function such as DNA replication or glycolysis use chemical specificity to keep their processes separate from other functional modules (Hartwell et al., 1999). It has been proposed recently that the future of genome design may be in minimal cells, combined with different functional modules to create cells for specific purposes (Gibson, 2014). This would require a detailed understanding of not only how a genome maps to its phenotype and how the genes themselves can form functional modules but also concerning the ways in which these modules interact. This is one of the main advantages of using a whole-cell model rather than a constraint-based model—from observing the behaviour of reactions, we can see how other mechanisms in the cell (e.g., DNA production) are affected, which we would not in a constraint-based model.

The metabolism submodel in the *M. genitalium* model is a central hub of activity and an integral stepping stone for substance transfer between cellular processes. Although internal mechanisms and local rules for the model were gathered from experimental data and are biologically valid, the complexity that arises from so many parameters being integrated together means that the model has to be treated as a black box. Analysing the behaviour of the model could ultimately lead to better biological understanding of the connections between cellular processes. If the way that two processes are coupled together *in silico* in the whole-cell model yields output that matches experimental data, this can help to develop insight into how these processes are linked in a real cell. This could aid genome design, where insights from modelling can rationally guide *in vitro* experiments and gene editing (Landon et al., 2019; Rees-Garbutt et al., 2020, Rees-Garbutt et al., 2021).

We can see from Figure 3 that the knockouts that cause DNA and septum disruptions cause similar behaviours in the flux profiles to non-essential gene knockouts, likely because most of their reaction behaviours were classified as normal. Supplementary Figure S3 shows that fewer than 10 reactions were consistently affected across the simulations within these phenotypic classes, so we can infer that these reactions might be the bridge between the metabolism process and the DNA replication or cytokinesis process. Limitations of the *M. genitalium* model mean that the results presented here do not include multiple cell divisions, and it is possible that more widespread effects on metabolism would be revealed in future work with more generations.

The interactions between the metabolism and the other phenotypic classes (protein and RNA) are less simple, as there are significantly more reactions that are consistently behaving abnormally. This is not surprising, as there are two main functions for a cell to perform: growth and replication. Growth occurs consistently through the cell cycle and requires constant synthesis and degradation of different proteins and RNAs. There is also a temporal element, as cascades of reactions that form different proteins may need to occur in a specific order. Any disruption to an aspect of this process during the life cycle will filter down to other processes, whereas if DNA replication is disrupted, it is primarily cell division that will be halted. In future studies, it would be interesting to see if dividing the proteins into functional groups and pathways for further analysis leads to a better understanding of their roles and how they interact with each other.

It is hard to draw solid conclusions about cell behaviours, as *M. genitalium* is an organism where not all of the genes are classified, and the data that the model was built upon are from many different sources and organisms. In terms of the network analysis, there are some reactions that have been observed in *M. genitalium* but do not have known enzymes to catalyse them, which leaves gaps within the model. There may be unexpected and unusual behaviours that are not captured in the training data as well, leading to misclassifications; for example, the reactions that performed badly in the neural network classifications may be sensitive to small changes in the metabolic network, meaning that their behaviours are inconsistent and unpredictable. However, it is useful to flag these reactions and, in the future, to use different approaches to understand their behaviours. There is also the possibility that, after applying the machine learning processes, the results show more about the internal features of the model itself than the actual biology, which is a good starting point for lab work.

As whole-cell models become more widely used, analysis software will become more important. The most recent whole-cell model is of *E. coli* (Macklin et al., 2020), which is a better-understood organism than *M. genitalium*, with significantly more data available to validate and add to it, so this is an important development for the field. However, the complexity of models will increase hugely with the size of the genome of the organism, and as *E. coli* has an order of magnitude more genes than *M. genitalium* (Blattner et al., 1997), analysis tools that can provide data processing and dimensionality reduction will be even more important for enhancing understanding and ultimately genome design.

## Data Availability

The datasets generated and analysed for this study can be found in the University of Bristol data repository https://doi.org/10.5523/bris.3u1v7dy42fk332watjl81m13y0. The code used for this publication can be found at https://doi.org/10.5523/bris.879w4p1r8iy32ef9vcwftq9ts.
