# Supplementary material for: Understanding Metabolic Flux Behaviour in Whole-Cell Model Output
Source: Front Mol Biosci. 2021 Dec 17;8:732079. doi: 10.3389/fmolb.2021.732079 (PMC8718694; doi:10.3389/fmolb.2021.732079)
Supplement: Supplementary file 1 [file DataSheet1.pdf]

SUPPLEMENTARY INFORMATION

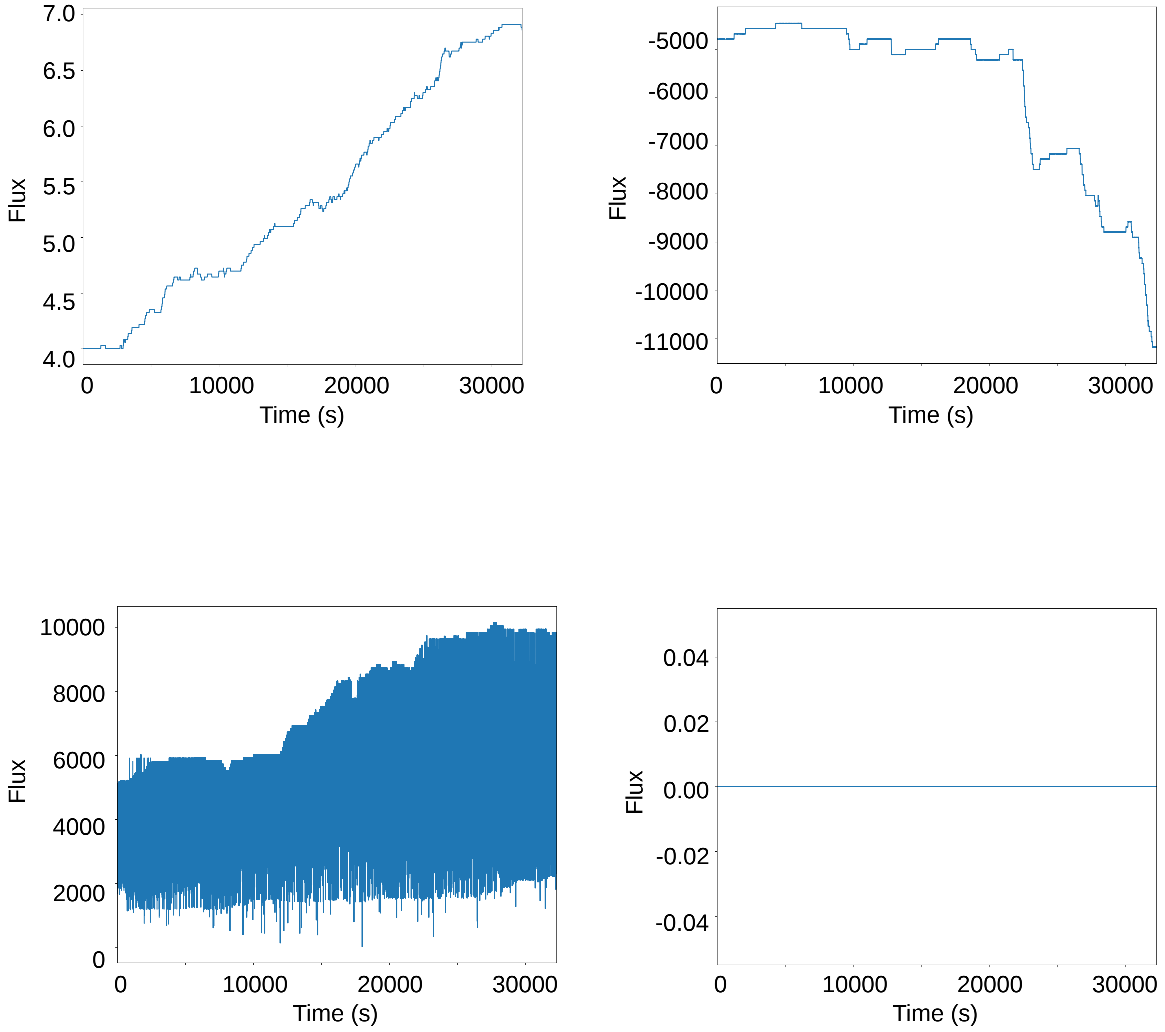

Figure S1. Examples of different metabolic flux behaviours, referred to by their reaction identifiers from the model (clockwise from top left: increasing (Pyk_GDP), decreasing (DcdK), stationary (TX_m1dG) and oscillatory (AceE)) for different reactions across the entire timeseries of a single simulation.

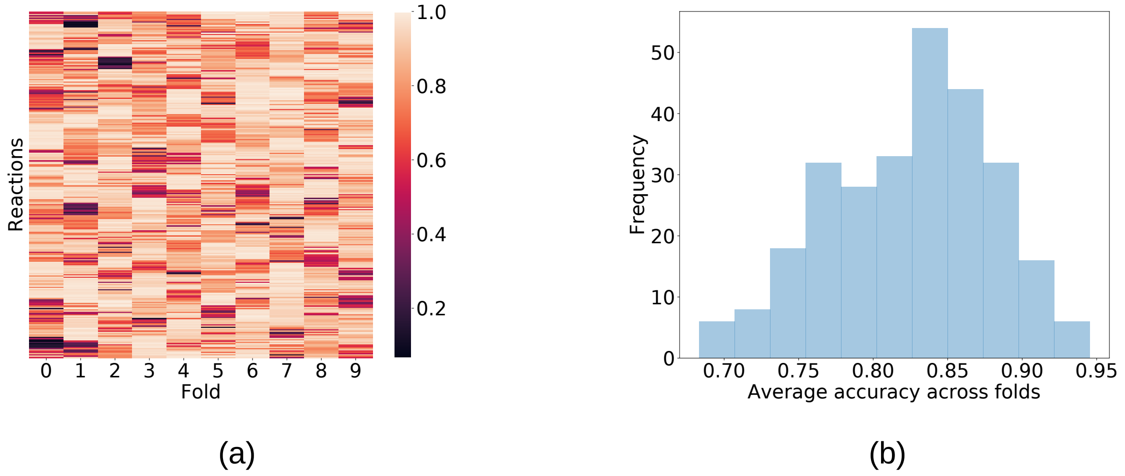

Figure S2. Heatmap of the accuracies of different folds after k-fold cross validation was carried out, for all reaction neural networks in a, and accuracies averaged across 10 folds of cross validation for each reaction neural network in b.

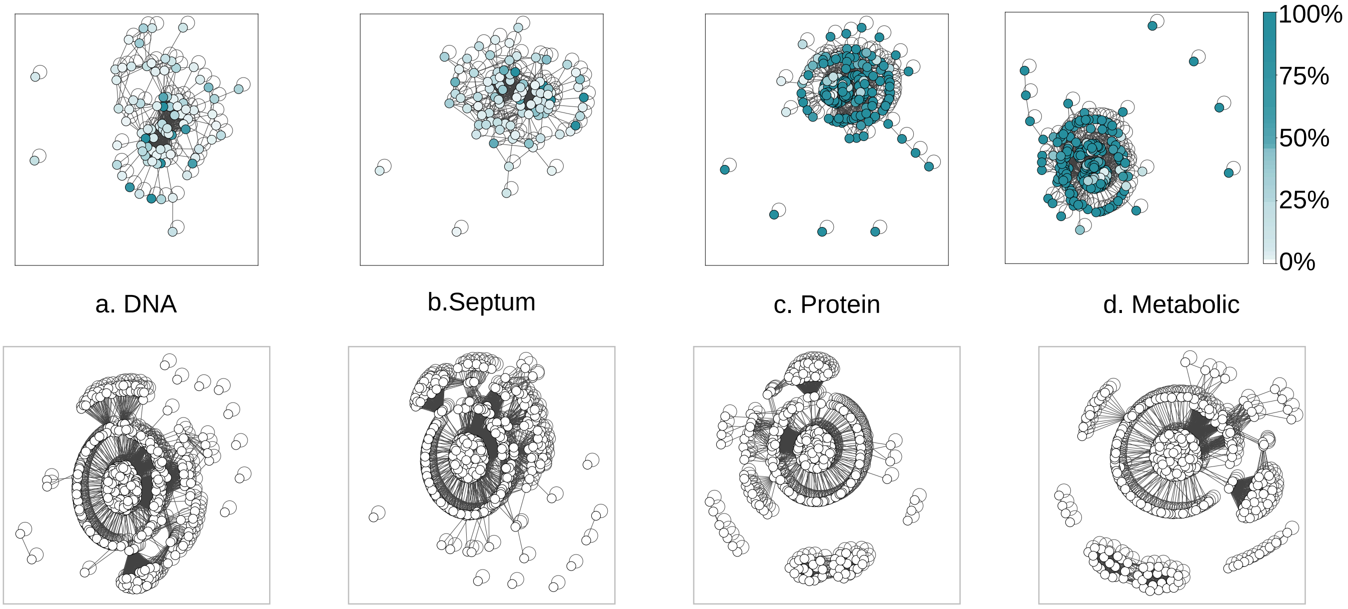

Figure S3. Sub-networks of the behaviour of reactions across the simulations within individual classes (shown here are the DNA, Septum, Protein and Metabolic phenotype classes). The reactions that are consistently behaving abnormally are shown on the top row, with a colour gradient that corresponds to how frequently the reaction behaves abnormally, and the remaining reactions (either those that behave normally, or are unclassified) are shown on the bottom row.

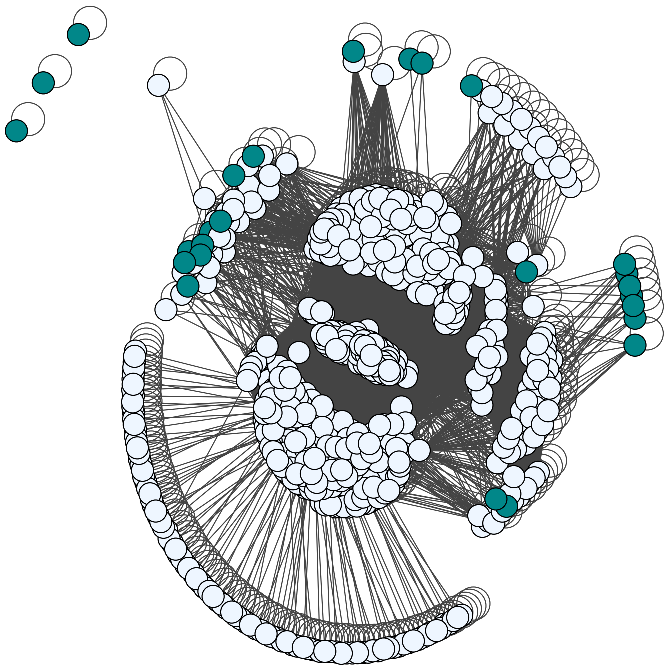

Driver Node
TXPYDX (Pyroxidal)
TX_ACAL (Acetaldehyde)
TX_CAP (Carbamoyl phosphate)
TX_CO2 (Carbon dioxide)
TX_COA (Coenzyme A)
TX_FOR (Formate)
TX_H202 (Hydrogen peroxide)
TX_HDCA (Hexadecanoate)
TX_HDCEA (Hexadecenoate)
TX_DDCA (Dodecanoate)
TX_LIPOATE (Lipoate)
TX_NAC (Nicotinade)
TX_O2 (Oxygen)
TX_OA (Oxaloacetate)
TX_OCDCA (Octadecanoate)
TX_OCDCEA (Octadecenoate)
TX_RIBFLV (Riboflavin)
TX_THF (Tetrahydrofolate)
TX_TTDCA (Tetradecanoate)
TX_TTDCEA (Tetradecenoate)
Upp (Uracil dephosphorylation)

Figure S4. The metabolic network shown in graph form, where the nodes are reactions (referred to by their reaction identifiers from the model) and edges are substrates that connect them (e.g. if a substrate is involved in two different reactions, this becomes an edge between the two reaction nodes). The driver nodes are shown in dark turquoise, which are the set of nodes that need to be controlled in order to have full control over the network. The driver nodes are listed in the adjacent table.
